# Lymphocyte activation gene-3 (LAG3) mRNA and protein expression on tumour infiltrating lymphocytes (TILs) in oesophageal adenocarcinoma

**DOI:** 10.1007/s00432-020-03295-7

**Published:** 2020-06-26

**Authors:** Florian Gebauer, Max Krämer, Christiane Bruns, Hans A. Schlößer, Martin Thelen, Philipp Lohneis, Wolfgang Schröder, Thomas Zander, Hakan Alakus, Reinhard Buettner, Heike Loeser, Alexander Quaas

**Affiliations:** 1 Department of General, Visceral, Tumour and Transplantation Surgery, University of Cologne, Kerpener Strasse 62, 50937 Cologne, Germany; 2 Institute of Pathology, University of Cologne, Cologne, Germany; 3 Department I of Internal Medicine, Centre for Integrated Oncology Aachen Bonn Cologne Duesseldorf, Gastrointestinal Cancer Group Cologne GCGC, University of Cologne, Cologne, Germany; 4 Centre for Molecular Medicine Cologne, University of Cologne, Cologne, Germany

**Keywords:** LAG3, Immunohistochemistry, mRNA in-situ, Oesophageal adenocarcinoma

## Abstract

**Purpose:**

Lymphocyte activation gene-3 (LAG3) is an immunosuppressive checkpoint molecule expressed on T cells. The frequency and distribution of LAG3 expression in oesophageal adenocarcinoma (EAC) is unknown. Aim of the study was the evaluation and distribution of LAG3 on tumour infiltrating lymphocytes (TILs) and correlation with clinico-pathological and molecular data.

**Methods:**

We analysed tumor tissue samples using immunohistochemistry, multi-colour immunofluorescence and mRNA in-situ technology. The analyses were performed on a multi-spot tissue microarray (TMA) with 165 samples, followed by an evaluation on a single-spot TMA with 477 samples. These results were correlated with clinical and molecular tumour data.

**Results:**

LAG3 expression on TILs was detectable in 10.5% on the multi-spot TMA and 11.4% on the single-spot TMA. There was a strong correlation between protein expression and mRNA expression (*p* < 0.001) in TILs. LAG 3 expression was correlated with CD4+ and CD8+ T-cells within the tumor (*p* < 0.001). LAG3 expression showed an improved overall survival (OS) compared to patients without LAG3 expression (median OS 70.2 vs. 26.9 months; *p* = 0.046). The effect was even clearer in the group of patients with tumour stages > pT2 (70.2 vs 25.0 months; *p* = 0.037).

**Conclusion:**

This is the first description of LAG3 expression on TILs in EAC, underscoring the importance of immunomodulation in EAC. Our data suggest an impact of LAG3 in a relevant subset of EAC. Therapeutic studies investigating the efficacy of LAG3 inhibition in EAC will also provide predictive evidence and relevance of the immunohistochemical determination of LAG3 expression.

## Introduction

Immunotherapy has grown to a rapidly advancing sphere of research on modern strategies for the treatment of cancer (Fridman 2017). A number of patients have already benefited from immune checkpoint blockades, and various drugs are currently under evaluation in clinical trials (Zhao and Subramanian 2018). In oesophageal adenocarcinoma (EAC)—a rapidly increasing cancer entity with a worse prognosis—surgery, chemotherapy and radiation remain the basis of treatment of EAC (Gore 2005; Grierson et al. 2017). The role of evolving immunotherapy has yet to be examined for EAC.

Immune checkpoints are a well-known form of immunomodulation, leading to a down-regulated immune response in the tumour microenvironment. Several of these checkpoints have been detected, such as programmed death cell protein 1 (PD-1), which is common and has occurred during the treatment of melanoma, non-small-cell lung cancer (NSCLC), renal cell carcinoma and urothelial carcinoma (Andrews 2017; Roberts 2017; Ma 2017). In addition to PD-1, lymphocyte activation protein-3 (LAG3) presents a targetable checkpoint, as can contribute to therapy strategies, including treatment of EAC.

LAG3 belongs to the immunoglobulin superfamily (IgSF) and is particularly displayed on several forms of T-lymphocytes (CD4+, CD8+, regulatory T-cells [Treg], tumour infiltrating lymphocytes [TILs]), as well as B-lymphocytes and dendritic cells (He 2016). LAG3 shares approximately 20% identity with the CD4 gene, and it binds to major histocompatibility complex 2 (MHC II) with greater affinity than CD4 (He 2016). The LAG3/MHC II complex on CD4+ cells negatively modulates T-cell activity and enhances antigen self-tolerance when displayed on CD8+ cells. Conversely, LAG3 binding to MHC II on Treg cells advances the suppressive effect on T-lymphocytes, enforcing the negative immune regulation effect of LAG3 (Andrews 2017). Studies have suggested that LAG3 is a negative regulator of T-cell activation and function, since the blockade of LAG3 on human CD4 clones resulted in enhanced proliferation, with an elevated production of IL-2, IL-4, IFN-γ and TNFα (Previte, et al. 2019; Goldberg and Drake 2011).

Analysis of LAG3 overexpression on TILs has revealed evidence for a pathological role, which involves down-regulating the immune response for various cancer entities, such as chronic lymphatic leukaemia, colorectal cancer, ovarian cancer, melanoma and hepatocellular carcinoma, leading to a worse prognosis in LAG3 positive malignancies (Shapiro 2017; Li 2013; Hemon 2011; Huang 2015; Chen and Chen 2014). Interestingly, and in contrast to these findings, recent studies in breast cancer patients showed a favourable outcome in LAG3 positive tumours regarding the overall survival (OS) of the patients, while other studies showed a worse prognosis in breast cancer (Sidaway 2017; Burugu 2017).

The aim of the present study is to assess the expression of LAG3 on TILs at the protein level, as well as the mRNA level, in EAC and correlate the expression profile with clinico-pathological and molecular data and the prognosis of individual patients.

## Material and methods

### Patients and tumour samples

The formalin-fixed and paraffin embedded samples from 477 patients with EACs, who underwent primary surgical resection or resection after neoadjuvant therapy between 1999–2014 at the Department of General, Visceral and Cancer Surgery, University of Cologne, Germany, were analysed. The standard surgical procedures were laparotomic or laparoscopic gastrolysis and right transthoracic en-bloc oesophagectomy, with intrathoracic oesophagogastrostomy, including two-field lymphadenectomy of mediastinal and abdominal lymph nodes, transhiatal extended distal oesophagectomy with transabdominal intrathoracic or cervical anastomosis as described previously (Holscher 2007). Patients with advanced oesophageal cancer (cT3), or lymph node metastasis in clinical staging, received preoperative chemoradiation (5-Fluouracil, cisplatin, 40 Gy) or chemotherapy. Follow-up data were available for all patients. Patient characteristics are given in Table 1. Depending on the effect of neoadjuvant chemo- or radiochemotherapy, there was a preponderance of minor responders, defined as having a histopathological residual tumour of ≥ 10% (Schneider 2008).

**Table 1:** Baseline characteristics of the entire patient cohort

	Total	Percent (%)
Sex		
Female	42	10.0
Male	379	90.0
Age group		
≤ 65	221	52.4
> 65	200	47.6
Tumour stage		
pT 1	50	11.9
pT 2	36	8.6
pT 3	322	76.8
pT 4	11	2.6
Lymph node status		
pN 0	161	38.3
pN +	259	61.7

According to the suggestions of the international immuno-oncology working group for assessing TILs on solid tumours, we constructed a multi-spot tissue microarray (TMA) (Simon et al. 2005; Helbig 2016; Hendry et al. 2017a, b). Construction of the multi-spot TMA and immunohistochemical staining procedures were performed as previously described (Simon et al. 2005; Helbig 2016). In brief, tissue cylinders, with a diameter of 1.2 mm each, were punched from selected tumour tissue blocks using a self-constructed, semi-automated precision instrument and embedded in empty recipient paraffin blocks. For the multi-spot TMA (165 patients), up to 8 tumour spots were punched out of the tumour, 4 spots each from the surface and the invasion front. The 165 patients evaluated using the multi-spot TMA were used as a test cohort. These data were statistically correlated with survival and molecular data, such as *TP53* mutational status.

We analysed 312 additional patients using a single-spot TMA. For this TMA, one tissue core from each tumour was randomly punched out and transferred into a TMA recipient block. 4 μm sections of the resulting TMA blocks were transferred to an adhesive coated slide system (Instrumedics Inc., Hackensack, NJ) for immunohistochemistry (IHC). All procedures performed for studies involving human participants were in accordance with the ethical standards of the institutional research committee and with the 1964 Helsinki Declaration, and its later amendments, or comparable ethical standards.

### Immunohistochemistry

IHC was performed on the TMA slides. The following antibodies were used for IHC studies: a rabbit IgG monoclonal antibody (clone D2G40; dilution 1:300; Cell Signaling Technology, Netherlands) was used for LAG3, a rabbit monoclonal antibody (clone SP7; dilution 1:50; Thermo Fisher Scientific, USA) was used for CD3 and a mouse monoclonal antibody (clone C8/144B, dilution 1:200; Dako/Agilent, USA) was used for CD8. All immunohistochemical staining was performed using the Leica BOND-MAX stainer (Leica Biosystems, Germany) according to the protocol of the manufacturer. The evaluation of immunohistochemical expression was assessed manually by two pathologists (AQ and HL). Discrepancies in the results, which occurred only in a small number of samples, were resolved by consensus review.

Multicolour immunohistochemical stainings were performed on a Ventana Discovery Ultra automatic staining system (Ventana/Roche, Basel, Switzerland) using following primary antibodies: rabbit anti-LAG3 IgG monoclonal antibody D2G40, mouse anti-CD8 monoclonal antibody C8/144B, mouse anti-FOXP3 monoclonal antibody 236A/E7 (Abcam, UK; dilution 1:100), rabbit anti-CD4 monoclonal antibody 4B12 (Roche, Switzerland, ready to use). After conjugation with an antibody-bound enzyme (horseradish peroxidase or alcalic phosphatase), detection was carried out using DISCOVERY Silver kit (LAG3), DISCOVERY Yellow kit (FOXP3), DISCOVERY Teal kit (CD8), DISCOVERY Red Kit (CD4; all Ventana/Roche, Switzerland)). Counterstaining was done with hematoxylin and bluing reagent.

### Strategy of evaluation

LAG3: < 1% of lymphocytes was defined as negative, 1–2% of lymphocytes were assessed as “LAG3 low”, > 2% of lymphocytes was counted as “LAG3 high”. The reading strategy followed the assessment of LAG3 in clinical trials in malignant melanoma, where the response rates of the LAG3 blockade correlated with LAG3 expression of ≥ 1% (Ascierto and McArthur 2017). For statistical analysis, the cut off was determined as ≥ 1%, thus low and high LAG3 expression was assessed as positive and < 1% expression as negative.

CD3: CD3 expression in < 3 lymphocytes/mm^2^ was evaluated as negative, > 3–50 lymphocytes/mm^2^ were assessed as low positive and > 50 lymphocytes/ mm^2^ were defined as high positive, considering peritumoral and intratumoral distribution.

CD8: CD8 was analysed according to the CD3 evaluation criteria. For statistical analysis, high expression of CD3 or CD8 with > 50 lymphocytes/mm^2^ were assessed as positive.

Regarding the multi-spot TMA considering eight tumor spots in total, four spots each of the tumour surface and the invasive margin, were examined. We calculated the average of the scores and matched the four samples to one category based on limit values: 0, negative; 0–0.9, low; 1–2, high (e.g. LAG3 expression in spot 1 = 2, spot 2 = 1, spot 3 = 0, spot 4 = 2, average of the spots: 1.25 → category “high”). Discrepancies in the results were resolved by consensus review.

### Immunofluorescence multi-colour staining

Immunofluorescence staining was performed on TMAs and whole section slides. Therefore, paraffin sections were deparaffinised and antigens were retrieved with EDTA at pH 8 (PT Module, Lab Vision Thermo Scientific). Slides were blocked using normal horse serum, for 30 min at room temperature (Vector Laboratories). Slides were incubated overnight at 4 °C with a master mix containing the primary antibodies (LAG3, 1:75, Cell Signaling; CD4, mouse monoclonal 4B12, 1:75, Thermo Fisher Scientific; CD3, rat monoclonal CD3-12, 1:50, Abcam; CD8, 1:100, Dako/Agilent). Slides were washed and stained with a master mix containing the corresponding secondary antibodies coupled to Alexa Fluor 555 (donkey anti-rabbit, Abcam), Alexa Fluor 594 (donkey anti-rat, Jackson Laboratories) and Alexa Fluor 647 (donkey anti-mouse, Jackson Laboratories) for 1 h at room temperature. Nuclei were visualised with DAPI (Sigma-Aldrich). Slides were mounted using an antifade solution (ProLong Diamond, Invitrogen) and scanned with a 40× objective (gSTED super-resolution confocal microscope, Leica). Images were adjusted for brightness and contrast using ImageJ (FIJI).

### mRNA in-situ (RNAScope)

The RNAscope assay was performed according to manufacturer’s instructions (Bott et al. 2011). In brief, paraffin-embedded TMA blocks were cut into 5 μm sections, pre-treated according to an extended protocol (30 min for pre-treatment 2 and 3), digested and hybridised at 40 °C in the HybEZ oven with the human LAG3 mRNA probe, which was provided by Advanced Cell Diagnostics Europe. The samples were then incubation with haematoxylin for 10 s. Target expression was compared to both negative (dapB) and positive (PPIB) controls. The scoring of the signals was performed as recommend by the manufacturer, where no staining or less than one molecule per 10 cells, score 0; 1–3 dots/cell, score 1; 4–9 dots/cell, score 2; 10–15 dots/cell, score 3; > 15 dots/cell, score 4. The DapB score was 0 and the PPIB score was 2. Positivity was defined as a score > 0. The determination of protein expression using immunohistochemistry, as well as mRNA expression, for LAG3 was assessed independently.

### Analysis of TP53 mutation status

The TP53 status was evaluated by immunohistochemistry on the single-spot TMA. The results were correlated with the *TP53*-mutational status by parallel genomic sequencing. A detailed description of the analysis was described previously (Quaas 2019). In brief, tumor DNA extraction was followed by amplification with a customized GeneRead DNAseq Targeted Panel V2 (Qiagen, Hilden, Germany), library construction and quantification. Exons 5-8 of the *TP53 gene* were sequenced on the MiSeq (illumina, Berlin, Germany) with a variance-cutoff of 5%. The results were only interpreted if the coverage was > 200×.

### Statistical analysis

Clinical data were collected prospectively according to a standardised protocol. SPSS Statistics for Mac (Version 21, SPSS) was used for statistical analysis. Interdependence between staining and clinical data were calculated using the chi-squared and Fisher’s exact tests and displayed by cross-tables. Survival curves were plotted using the Kaplan–Meier method and analysed using the log-rank test. Univariate and multivariate analyses were performed for prognostic factors of overall survival using the Cox regression model. All tests were two-sided. *p *values < 0.05 were considered statistically significant.

## Results

### Patient baseline characteristics

Patient characteristics are given in Table 1. A total of 421 patients with EAC that underwent surgical tumour resection were immunohistochemically interpretable. Reasons for non-informative cases included lack of tissue samples or absence of unequivocal cancer tissue in the TMA spot. Operative procedures were either thoraco-abdominal en-bloc oesophagectomy (*n* = 269, 63.9%) with intrathoracic anastomosis or transhiatal oesophagectomy with transabdominal or cervical anastomosis (*n* = 152, 36.1%). For the single-spot TMA, 42 patients (10.0%) were female, 379 (90.0%) male. A similar distribution was found for the multi-spot TMA (9.7% female, 90.3% male). The median age of the entire patient cohort was 65.2 years (range 33.6–85.6 years) at the time of diagnosis. Neoadjuvant treatment (chemo- or radiochemotherapy) was administered to 271 patients (59.8%) before operation from the single-spot TMA samples and 23 patients (13.9%) from the multi-spot-TMA samples. The median follow-up for the entire cohort was 52.0 months.

### LAG3 protein and mRNA expression

**Fig. 1:**
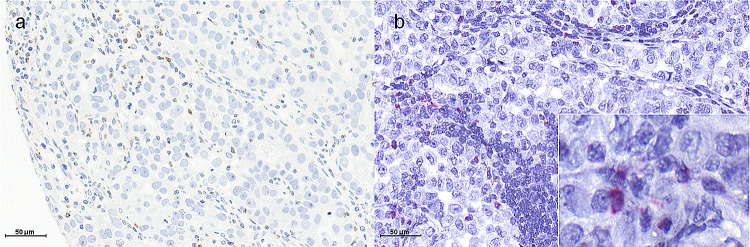
Immunohistochemistry and mRNA-Scope analysis of LAG3 in EAC. **a** Immunohistochemical LAG3 expression on TILs; **b** LAG3 mRNA expression on TILs (red signals)

LAG3 immunostaining was localised in the cytoplasm/membrane of tumour infiltrating lymphocytes (Fig. 1). LAG3 expression of ≥ 1% was assessed as positive. In total, on the single-spot TMA, LAG3 positivity was detectable in 11.4% (*n* = 48) of interpretable EAC cases and 10.5% (*n* = 17) of the multi-spot TMA samples (Table 2). The latter demonstrated a heterogenic LAG3 distribution within the four spots of each patient, the invasive and the surface tumour margin, in 60.1% (*n* = 100). However, comparing the expression pattern of the surface with the invasive tumour margin, a low heterogeneity was observed, only one case (0.6%) was positive for LAG3 on the surface and not on the invasive margin.

**Table 2:** Correlation of clinico-pathological status and LAG3 expression of single-spot and multi-spot TMA

	LAG3 expression single-spot TMA						LAG3 expression multi-spot TMA					
		Negative		Positive		*p *value		Negative		Positive		*p *value
Total	421	373	88.6%	48	11.4%		161	140	84.8%	21	15.2%	
Sex												
Female	42	36	85.7%	6	14.3%	0.424	16	15	93.8%	1	6.3%	0.694
Male	379	337	88.9%	42	11.1%		145	125	86.2%	20	13.8%	
Age group												
< 65	221	189	85.7%	32	14.3%	0.546	70	65	92.9%	5	7.1%	0.060
> 65	200	179	89.4%	21	10.6%		90	74	82.2%	16	17.8%	
pT stage												
1	50	41	82.0%	9	18.0%	0.370	47	44	93.6%	3	6.4%	0.061
2	36	31	86.1%	5	13.9%		29	21	72.4%	8	27.6%	
3	322	290	90.1%	32	9.9%		82	72	87.8%	10	12.2%	
4	11	10	90.9%	1	9.1%		1	1	100.0%	0	0.0%	
pN stage												
0	161	135	83.9%	26	16.1%	0.018	61	55	90.2%	6	9.8%	0.352
pos	259	237	91.5%	22	8.5%		98	83	85.9%	15	14.1%	
Neoadjuvant therapy												
No	165	148	89.7%	17	10.3%	0.639	139	121	87.1%	18	12.9%	1.000
Yes	256	225	87.9%	31	12.1%		22	19	86.4%	3	13.6%	

LAG3 mRNA expression was analysed in 77 patients from the multi-spot TMA and positive in 36 patients (46.8%) on the surface margin and in 28 patients (35.9%) on the infiltration margin. Compared to detection by IHC, LAG3 mRNA expression showed higher expression frequencies on both the surface and infiltration margins. Despite the higher total number of LAG3 positive samples, as determined by mRNA base scope, there was a strong correlation between IHC and mRNA detection (*p* < 0.001). On the surface margin, 77.1% of the LAG3 IHC negative patients showed no mRNA expression, and similar results were found for the infiltration margin (85.7%). No patient in any of the groups exhibited detectable protein expression without the presence of mRNA expression. No correlation between TP53 mutations, HER2 overexpression and LAG3 expression was revealed (*p* = 0.383 and *p* = 1.000, respectively).

### CD3 and CD8

**Fig. 2:**
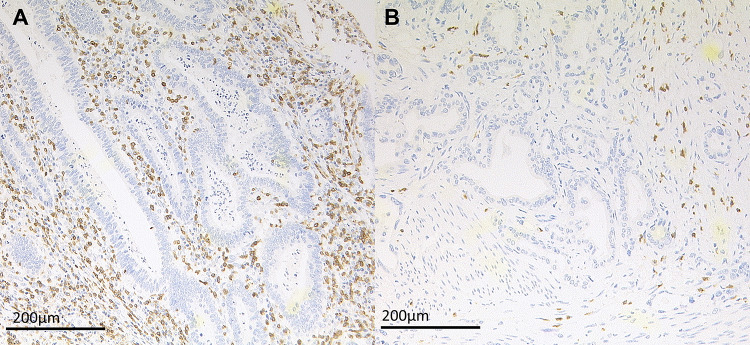
Immunohistochemistry of CD3 in EAC. **a** High expression of CD3 with > 50 positive TILs/mm^2^; **b** low expression of CD3 positive TILs

The status of CD3 was evaluated on single- and multi-spot TMA; CD8 infiltrating T-cells were only evaluated on the multi-spot TMA. CD8 demonstrated a similar distribution pattern as CD3, but to a lesser degree. High amounts of CD3 TILs were associated with an improved OS compared to CD3 poor tumours (Fig. 2a). In both tumour regions, roughly half of the tumours presented with a high accumulation of CD3 TILs (luminal 49.1%, invasive front 51.5%), which was correlated in the cross-table analysis (*p* < 0.001). There was no difference between the surface and the invasive tumour margin with respect to the amount of CD3 TILs. CD3 distribution within the tumour was predominantly peritumoral (*n* = 130; 78.8%) and showed no difference between the surface and the invasive tumour margin. High amounts of CD3 and CD8 TILs featured a strong correlation with high LAG3 expression (*p* < 0.001).

### LAG3 and co-expression of T-cell subset markers

**Fig. 3:**
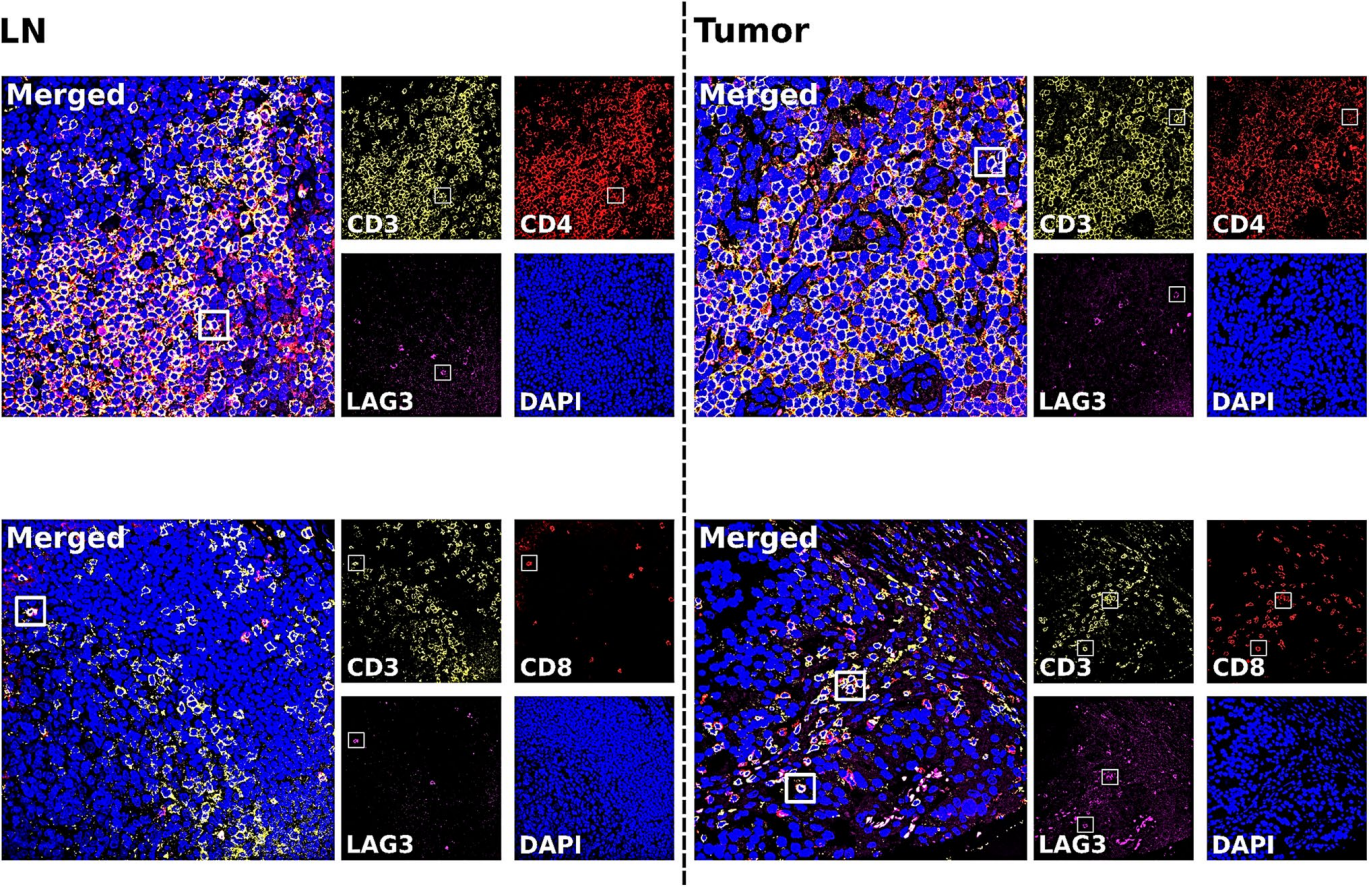
Immunofluorescence of multicolour staining for LAG3 (purple signals), CD3 (yellow signals), CD4 and CD8 (red signals) and counterstaining of the nuclei with DAPI (blue signals). **a**, **b** Show normal lymph node tissue; in **a** with high expression of CD3 and CD4 and single-cell co-expression with LAG3; in **b** single cells positive for LAG3 co-expressed CD3 and CD8. **c**, **d** Show tumour tissue with surrounding immune cells; in **c** the co-expression of LAG3 with CD3 and CD4 is seen; in **d** LAG3 positive cells are positive for both CD3 and CD8

**Fig. 4:**
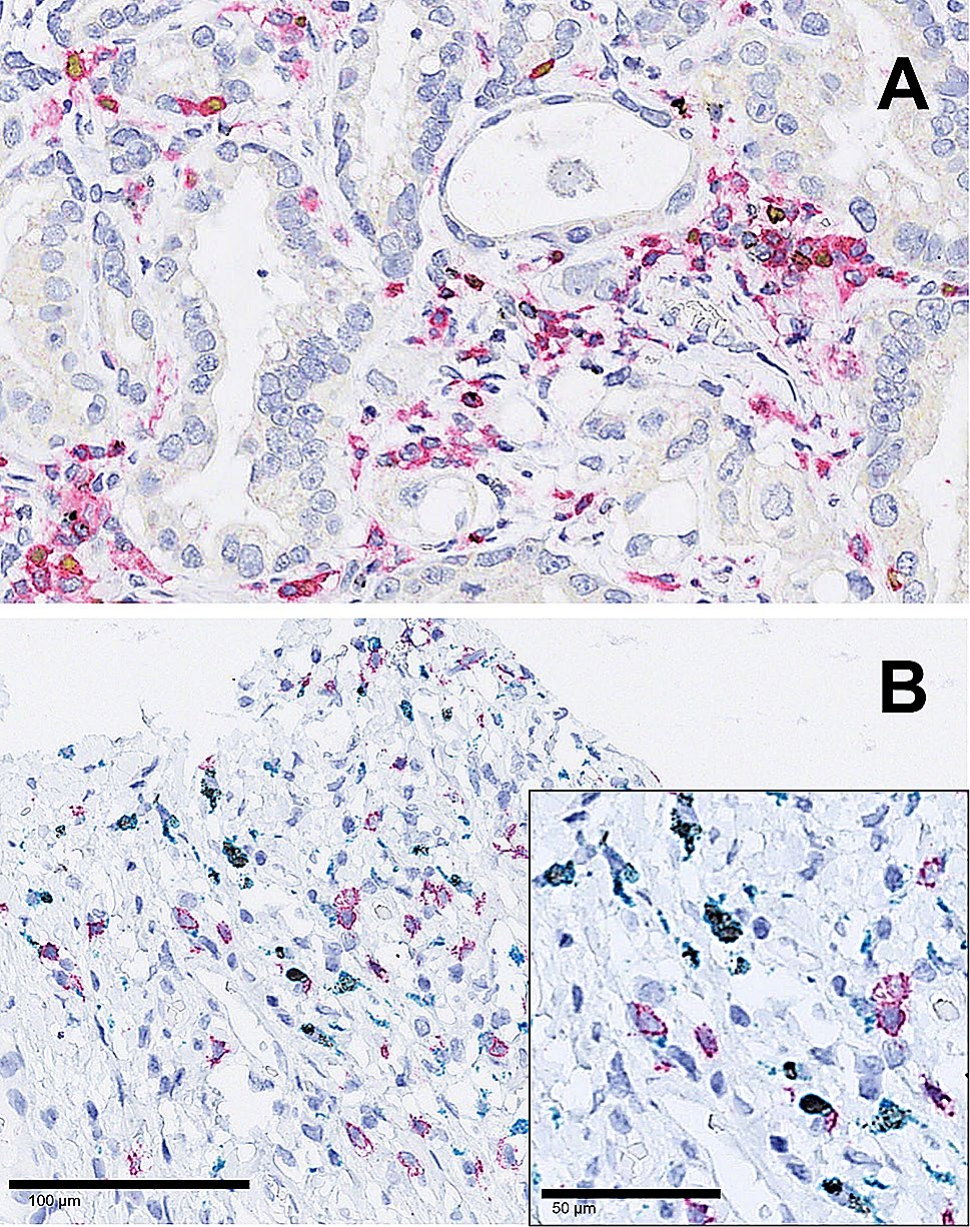
Multicolour IHC for LAG3 (black signals), CD4 (red signals), FOXP3 (yellow signals) and CD8 (blue signals): **a** co-expression of LAG3, CD4 and FOXP3 in a minor fraction; **b** predominant co-expression of LAG3 with CD8 (inserted detail), here no relevant co-expression with CD4

To evaluate which subtypes of T-cells expressed LAG3, we performed multicolour immunofluorescence and multicolour immunohistochemistry staining on two exemplary TMAs. In a semiquantitative analysis, we correlated LAG3 positive cases with the expression of CD3, CD4 and CD8 (Figs. 3 and 4). In normal lymphatic tissue, LAG3 is co-expressed with CD3, CD4 CD8 and FOXP3. In EAC multicolour immunohistochemistry, a predominant co-expression of LAG3 and CD8 was seen, only a minor fraction demonstrated positivity for LAG3 and CD4 or FOXP3. In multicolour immunofluorescence LAG3 positive cells within the tumour microenvironment were seen with co-expression of CD3, CD4 and CD8.

### LAG3 as a prognostic biomarker

**Fig. 5:**
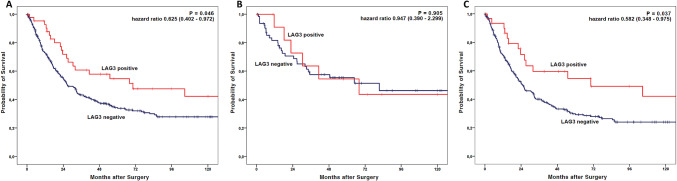
**a** Tumours with LAG3 positive TILs showed a better overall survival. 70.2 months (95% confidence interval (CI) 1.9–138.5 months) in LAG3 positive tumours compared to a median OS of 26.9 months (95%CI 21.9–31.8 months, *p* = 0.046) for LAG3 negative tumours. **b** In early invasive tumours, LAG3 dependent survival difference could not be revealed, the survival difference in the entire patients´ cohort is therefore driven by advanced tumour stages (< pT2) (**c**)

To analyse the impact of LAG3 expression on TILs, Kaplan Meier survival analysis were performed on the single-spot TMA. The OS in patients with LAG3 expression significantly improved compared to LAG3 negative tumours. The median OS was 70.2 months (95% confidence interval (CI) 1.9–138.5 months) in LAG3 positive tumours compared to a median OS of 26.9 months (95% CI 21.9–31.8 months, *p* = 0.046) in LAG3 negative cases (Fig. 5a). The effect was independent of whether neoadjuvant treatment was administered or not. The observed survival difference in the entire patients´ cohort is predominantly driven by advanced tumour stages (> pT2). LAG3 positive tumours with tumour stages > pT2 had a median OS of 70.2 months (95% CI 20.6–32.0 months), while LAG3 negative tumours with tumour stages > pT2 showed a median OS of 25.0 months (95% CI 2.1–154.6 months) (*p* = 0.037). In the group of pT1/2 tumours, a LAG3 dependent survival difference could not be revealed (Fig. 5b and c). In a multivariate cox-regression model, LAG3 expression alone failed to serve as an independent prognostic marker due its correlation with advanced tumour stages (Table 3).

**Table 3:** Multivariate analysis of clinico-pathological status and LAG3 expression for the entire patients´ cohort analysed on the single-spot TMA

	Significance (*p* value)	Hazard ratio	95% confidence interval	
			Lower	Upper
Sex (female vs. male)	0.023	1.742	1.081	2.808
Age group (< 65 vs > 65 years)	0.212	1.149	0.924	1.428
pT (pT1/2 vs pT3/4)	0.018	1.501	1.072	2.100
pN (pN0 vs pN +)	< 0.001	2.804	2.103	3.739
LAG3 (pos. vs neg.)	0.312	0.837	0.593	1.182

## Discussion

In a large set of 421 patients with EAC, we report the impact of the checkpoint inhibitor LAG3 considering the protein and mRNA expression, as well as the distribution pattern within the tumour, in correlation with clinical and molecular data. In our cohort, LAG3 was positively correlated with subset of T-cells (predominant CD8 positive T-cells). Additionally, elevated LAG3 expression was linked to a significantly better outcome for patients in the advanced tumour stages subgroup.

The construction of the multi-spot TMA for the analysis of tumour heterogeneity and associated TILs followed the recommendations of the International Immuno-Oncology Biomarkers Working Group (Hendry et al. 2017a, b). We started our analysis with a test-cohort of 165 patients using the multi-spot TMA, followed by a much larger cohort considering additional patients to reassess the results found before. The multi-spot TMA demonstrated a heterogenic LAG3 distribution within the eight tumour spots; however, by comparing the tumour surface with the infiltration margin, we found a consistent expression pattern of LAG3. Thus, in the case of endoscopically taken biopsies LAG3 expression in EAC can serve as a reliable predictor regarding the overall LAG3 expression within the tumour.

In contrast to our initial hypothesis, we are able to show a favourable outcome of patients with elevated LAG3 in TILs. However, most probably due to a correlation with pT-stage and lymph node metastasis, the LAG3 expression failed to serve as an independent prognostic marker, since the survival benefit correlated with LAG3 expression was predominantly detectable in pT3/4 tumours. This is fully in line with recent studies on breast cancer patients but in contrast to previous results in other solid tumour entities, such as melanoma and hepatocellular carcinoma (Li 2013; Hemon 2011; Sidaway 2017). However, divergent descriptions concerning the prognostic impact of a single immune checkpoint between different tumour entities are well known and likewise given for PD-1/PD-L1 (Bertucci 2015, 2017). This phenomenon can be explained by various analysis techniques and antibodies or by different organic systems. Due to technical variabilities, we analysed the mRNA expression profile of LAG3 for 77 patients and compared it to the protein expression detected using IHC in a double-blinded examination. Since there was a distinct correlation between the mRNA and protein expression profiles of LAG3, we confirm the viability of our antibody and analysis techniques.

The favourable outcome in context with LAG3 expression appears to be counterintuitive, initially, considering the suppressive effect of this immune checkpoint. Furthermore, our distinct results require exploratory approaches to clarify the role of immunomodulation in EAC. We, therefore, developed hypotheses to explain how LAG3 overexpression could be related to a better outcome.

An elevated expression may be part of a powerful immune reaction leading to an effective antitumoral response. Thus, we assume that pro-inflammatory signalling pathways against the tumour could be compensatory regulation by the suppressive effect of LAG3. We found a strong correlation between the existence of T-cells (CD3) and LAG3, supposing that an inflammatory microenvironment is attended by increased LAG3. Since tumours with elevated T-cells showed a significantly better outcome in our cohort, we underline the importance of an inflammatory antitumoral response, which is in accordance with former studies on EAC (Noble 2016). We therefore presume that LAG3 serves as a biomarker for a strong immune response. The initially assumed inhibitory immunomodulatory effect does not seem to be reflected by our current analysis to the extent expected. As described above, similar effects have been demonstrated for PD-L1 expression in different tumour entities. Whether a pharmacological LAG3 inhibition will prove to be effective in EAC cannot be conclusively answered on the basis of the available data. In our opinion, however, a positive effect of LAG3 inhibitor therapy can be assumed. However, further functional investigations with regard to LAG3 expression and interactions with tumour cells are necessary.

Taken together, we presume that LAG3 serves as a surrogate parameter in immunogenic tumour biology; although, we must admit that the limited knowledge of downstream mechanisms and the interaction with other intracellular pathways only allows us to hypothesise about a possible function of the protein in EAC. In addition, it remains obscure why only advanced tumour stages revealed a prognostic impact of LAG3 in contrast to early stages, underlining the urgent need for further research on immunomodulation.

Nevertheless, our assumptions support the use of LAG3 inhibition for EAC, since the loss of regulatory survival mechanisms would lead to a stronger immune response via CD3/8. Therapeutic studies investigating the efficacy of LAG3 inhibition in EAC will also provide predictive evidence on the determination of the LAG3 expression in the tumour microenvironment, as we have learned in the past for the determination of PD-L1 in lung cancer.

## Availability of data and material

The original data can be requested from the corresponding author, including SPSS tables and statistical syntax, immunohistochemical and rRNA data.

## Abbreviations

CI: Confidence interval
EAC: Oesophageal adenocarcinoma
IgSF: Immunoglobulin superfamily
IHC: Immunohistochemistry
LAG3: Lymphocyte-activation-protein 3
MHC: Major histocompatibility complex
OS: Overall survival
PD-1: Programmed death cell protein 1
TIL: Tumour infiltrating lymphocyte
TMA: Tissue microarrays
